# Birth of a child with trisomy 9 mosaicism syndrome associated with paternal isodisomy 9: case of a positive noninvasive prenatal test result unconfirmed by invasive prenatal diagnosis

**DOI:** 10.1186/s13039-015-0145-4

**Published:** 2015-06-26

**Authors:** Jingmei Ma, David S. Cram, Jianguang Zhang, Ling Shang, Huixia Yang, Hong Pan

**Affiliations:** Department of Obstetrics, Peking University First Hospital, No.1 Xianmen Street, Xicheng District, Beijing, 100034 China; Berry Genomics, Building 9, No 6 Court Jingshun East Road, Chaoyang District, Beijing, 100015 China

**Keywords:** Noninvasive prenatal testing, Prenatal diagnosis, Karyotyping, Fluorescent in situ hybridization, Quantitative fluorescent PCR, Trisomy 9 mosaicism syndrome, Isodisomy 9, Uniparental disomy

## Abstract

**Background:**

Non-invasive prenatal testing (NIPT) is currently used as a frontline screening test to identify fetuses with common aneuploidies. Occasionally, incidental NIPT results are conveyed to the clinician suggestive of fetuses with rare chromosome disease syndromes. We describe a child with trisomy 9 (T9) mosaicism where the prenatal history reported a positive NIPT result for T9 that was unconfirmed by conventional prenatal diagnosis.

**Methods:**

NIPT was performed by low coverage whole genome plasma DNA sequencing. Karyotyping and fluorescent in situ hybridization (FISH) analysis with chromosome 9p-ter and 9q-ter probes was used to determine the somatic cell level of T9 mosaicism in the fetus and child. Quantitative fluorescent PCR (Q-PCR) of highly polymorphic short tandem repeat (STR) chromosome 9 markers was also performed to investigate the nature of the T9 mosaicism and the parental origin.

**Results:**

A 22 month old girl presented with severe developmental delay, congenital cerebral dysplasia and congenital heart disease consistent with phenotypes associated with T9 mosaicism syndrome. Review of the prenatal testing history revealed a positive NIPT result for chromosome T9. However, follow up confirmatory karyotyping and FISH analysis of fetal cells returned a normal karyotype. Post-natal studies of somatic cell T9 mosaicism by FISH detected levels of approximately 20 % in blood and buccal cells. Q-PCR STR analysis of family DNA samples suggested that the T9 mosaicism originated by post-zygotic trisomic rescue of a paternal meiotic II chromosome 9 non-disjunction error resulting in the formation of two distinct somatic cell lines in the proband, one with paternal isodisomy 9 and one with T9.

**Conclusion:**

This study shows that NIPT may also be a useful screening technology to increase prenatal detection rates of rare fetal chromosome disease syndromes.

## Background

Prenatal diagnosis by chorionic villous sampling or amniocentesis is the gold standard method for detection of fetal chromosomal abnormalities during pregnancy. With the advent of next generation sequencing technologies, it is now possible to detect fetal chromosomal abnormalities by massively parallel sequencing of the cell free DNA in the maternal circulation which contains on average 10–20 % of fetal DNA during the second trimester [1]. Several large prospective trials [2, 3] have demonstrated that noninvasive prenatal testing (NIPT) is reliable and accurate with false positive and negative rates in the range of 0.1–0.2 %. Nonetheless, as the fetal DNA tested originates from the placental trophoblasts, it is strongly recommended that all positive NIPT results are confirmed by invasive prenatal testing [4]. While NIPT currently focuses on detection of common fetal autosomal aneuploidies such as T21, T18 and T13 as well as sex chromosomal aneuploidies, there has been research reports of positive results including T9 [5] and T2 [6], suggesting that NIPT may also have clinical utility for detection of rarer fetal trisomies. In this study, we present a confounding case of T9 mosaicism that was originally detected by NIPT, but was not confirmed by traditional invasive prenatal diagnosis methods.

## Case presentation

**Fig. 1:**
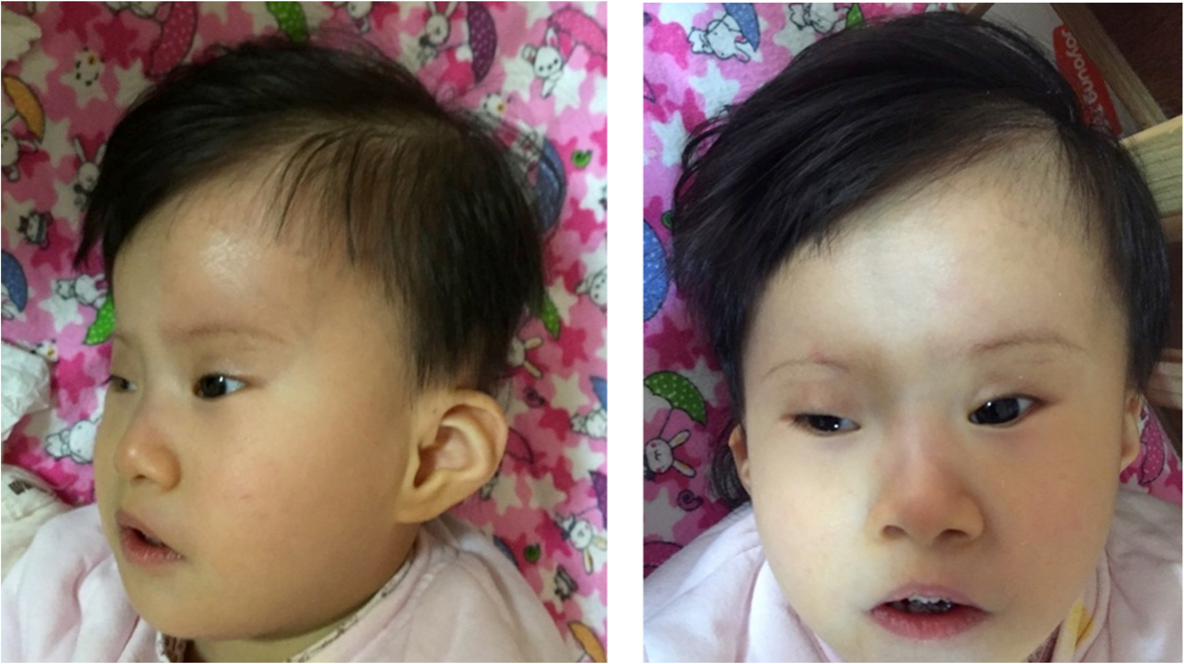
Dysmorphic craniofacial features of the proband with trisomy 9 mosaicism syndrome

A 22 month old girl presented at our genetic counselling clinic at the Peking University First Hospital with severe motor and intellectual disability, recurrent respiratory infection and failure to thrive. Physical examination revealed severe growth retardation with a weight of 7.5 kg (<3rd centile 10.9 kg), height of 62 cm (<3rd centile 88.9 cm) and head circumstance of 40 cm (<3rd centile 50 cm). She displayed moderate to severe hypertonia. Dysmorphic features were visibly evident, including microcephaly, craniofacial asymmetry, long face, ocular hypertelorism, short and upward slanting palpebral fissures, short frenulum of the upper lip, high arched palate, low-set ears and abnormal nasal features including a prominent nasal bridge with a short root, a small fleshy tip and slit-like nostrils (Fig. 1). X-ray examination also revealed scoliosis. At 22 months of age, her developmental milestones were only equivalent to normal children of 7–9 months of age. She could not follow obvious movements with her eyes and struggled to hold her head up. The parents indicated that she had not developed any form of speech.

**Fig. 2:**
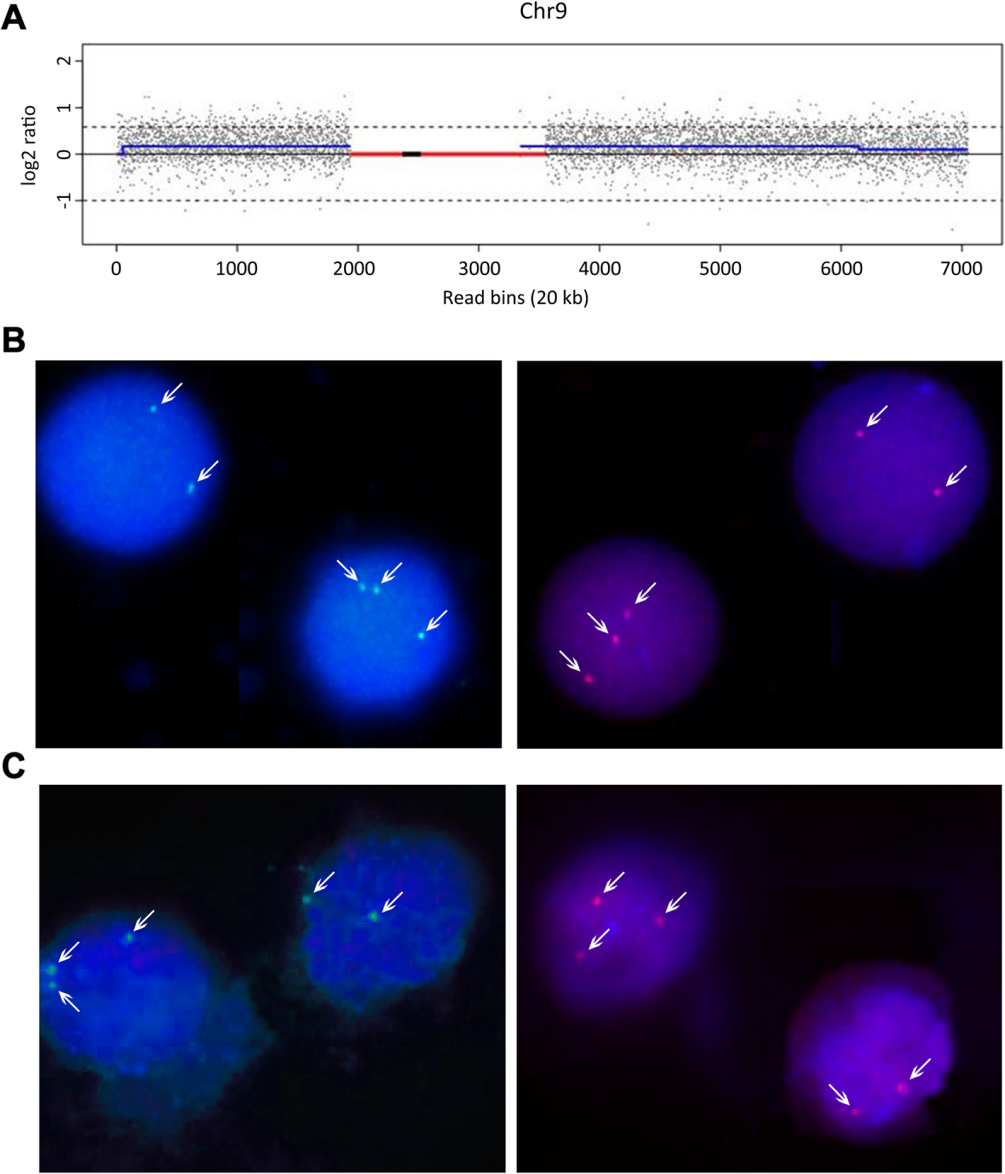
Levels of T9 mosaicism in somatic tissues. **a**. NIPT result for Chr9. The Chr9 plot shows the mean copy number variation per 20 kb sequencing bin (blue line) versus each 20 kb sequencing bin. The upper dashed line indicates the expected position of the blue line for 100 % T9. **b**. Analysis of white blood cells with fluorescein labeled 9p (green) and rhodamine labeled 9q (red) FISH probes. **c**. Analysis of white blood cells with 9p and 9q FISH probes. Representative FISH results (B and C) in a single viewing field are shown for two cells as examples of disomy 9 and T9. White arrows indicate the position of FISH signals for Chr9

In review of the pregnancy history, maternal serum screening at 15 weeks gestation revealed a risk for the Down Syndrome of 1 in 200. Based on this risk, the couple elected to have confirmatory NIPT at 16 weeks. Chromosome (Chr) z-scores were 0.62 (Chr21),−0.27 (Chr18) and 0.016 (Chr13), all within the normal z-score range (−3 < z < 3). However, the z-score for Chr9 (12.92) was outside the normal range, suggestive of T9. After calculation of copy number, the percentage of T9 was 30.1 % (Fig. 2a), suggesting T9 mosaicism. The couple’s clinician was informed about the possibility of a T9 mosaic fetus, prompting further prenatal investigation.

No structural anomalies in the second trimester were evident by ultrasound. However, at 30 gestational weeks, intrauterine growth restriction and lateral cerebral ventriculomegaly (right, 14 mm) was observed. An echocardiography revealed no cardiac abnormalities. Further invasive prenatal diagnosis by percutaneous umbilical blood sampling was therefore performed at 34^+2^ gestational weeks in order to confirm the suspected T9 mosaicism or the presence of other possible chromosomal abnormalities. The fetal karyotype determined by analysis of 20 metaphase cells was 46, XX. Testing of both parents revealed normal peripheral blood karyotypes. No further molecular genetic testing was undertaken.

The girl was born by vaginal delivery at 41 weeks with a birth weight of 1960 g, length of 45 cm and head circumstance of 30 cm (<5th centile 36 cm). The newborn was referred to the Pediatrics department. Following ultrasound and MRI assessment, the final clinical diagnosis was congenital cerebral dysplasia, intracranial hemorrhage, thrombocytopenia and congenital heart disease (ventricular septal defect, patent ductus arteriosus). Further genetic testing was recommended.

The parental guardians of the child provided written consent to conduct post-natal genetic testing of the child at 22 months of age. Peripheral blood karyotyping of 200 metaphase cells identified 199 cells with a normal 46, XX karyotype and one cell with an abnormal 47, XX,+9 karyotype. Based on guidelines and recommendations of the International System for Human Cytogenetics Nomenclature [7], this karyotype should normally be reported as 46, XX. To investigate the presence of T9 mosaicism further, analysis of cultured blood cells with chromosome 9p-ter and 9q-ter FISH probes detected T9 in 1 of 100 (1 %) cells examined, consistent with karyotyping. However, interphase FISH analysis with the same probes applied to an uncultured peripheral blood smear revealed T9 in 19 of 100 (19 %) cells examined (Fig. 2b). To confirm T9 mosaicism in another tissue type, interphase FISH was also used directly to assess cheek buccal cells. T9 mosaicism was found in 17 of 100 (17 %) cells examined (Fig. 2c). On this basis, interphase FISH suggested that the somatic level of T9 mosaicism was approximately 20 %, differing slightly from the level of 30 % in placental tissue detected by NIPT.

**Fig. 3:**
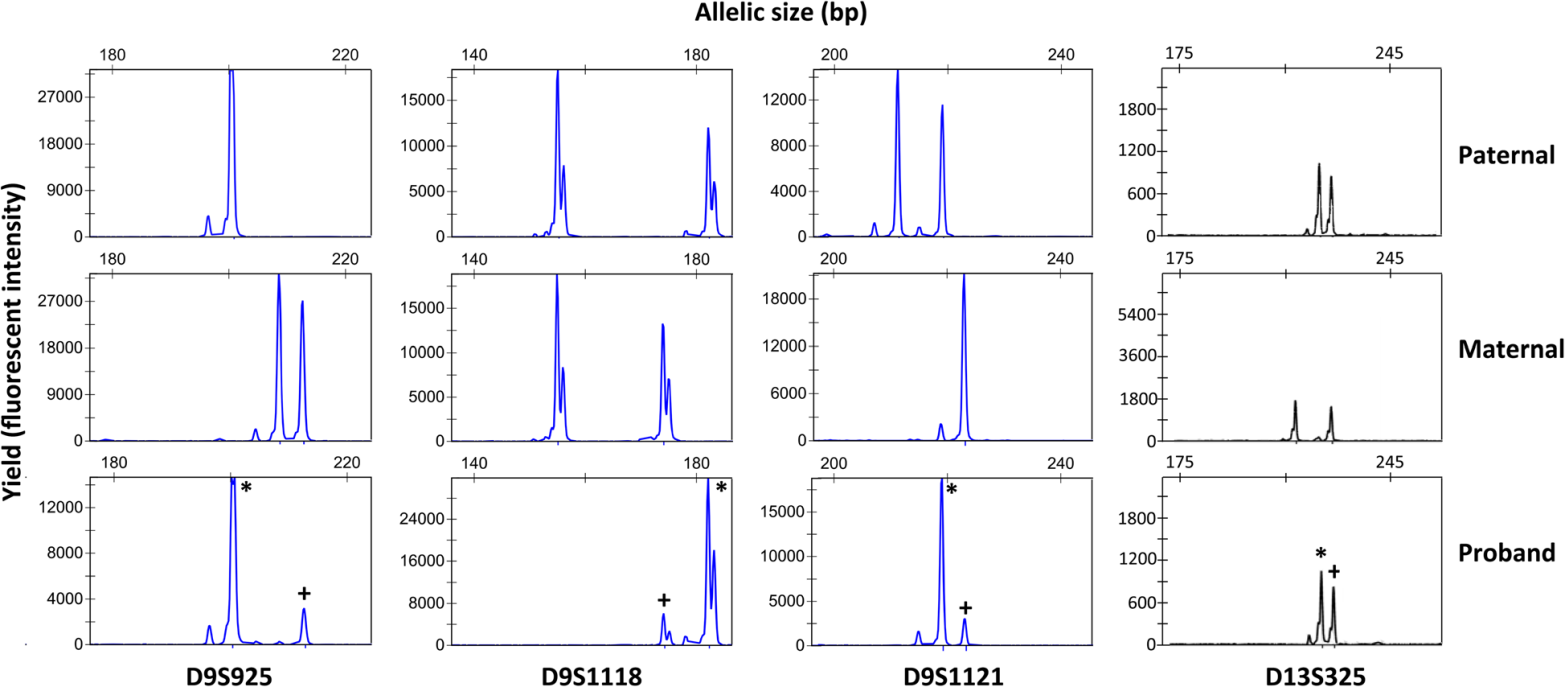
QF-PCR analysis of family genomic DNA samples for revealing the nature and origin of T9 mosaicism. Plots show yield of STR PCR products (Y-axis) versus allelic size (X-axis). Blue and black peaks represent 6-FAM and TAMRA labeled allelic products, respectively. The dominant paternal alleles (isodisomy 9) and the minor maternal alleles for informative STR markers D9S925, D9S1118 and D9S1121 that were inherited by the proband are denoted by symbols (*) and (+), respectively. Control marker D13S325 shows two allelic peaks indicating expected disomy 13

In order to investigate the nature of the T9 mosaicism and parental origin, we applied quantitative fluorescent PCR (QF-PCR) to analyse paternal, maternal and proband allelic patterns of short tandem repeat (STR) Chr9 markers. The alleles of STR markers D9S925, D9S1118 and D9S1121 were found to be informative for determining familial inheritance patterns (Fig. 3). All three Chr9 STR markers for the proband displayed a skewed bi-allelic pattern with allelic peak ratios of approximately 10:1 whereas the control D13S325 displayed a bi-allelic pattern with the expected peak ratio of approximately 1:1. Given that none of the Chr9 STR markers revealed a tri-allelic pattern typical of trisomy 9, we deduced that the dominant Chr9 STR allelic peaks were consistent with inheritance of two identical paternal alleles (isodisomy) whilst the minor Chr9 STR allelic peaks were maternally inherited. Further, since the expected allelic peak ratio for a full T9 displaying a bi-allelic marker pattern is 2:1, then the observed peak ratios of approximately 10:1 suggest a level of around 20 % T9 mosaicism, which was very similar to levels determined by FISH (Fig. 2b and 2c). Taken together, the proband inheritance pattern for Chr9 STR markers indicated the presence of two distinct cell lines in the child, one with paternal isodisomy 9 in approximately 80 % of somatic cells and T9 in the remaining somatic cells.

## Discussion

T9 syndrome and its related form T9 mosaicism syndrome are two extremely rare human chromosomal disorders. The first babies born with T9 [8] and T9 mosaicism [9] were reported in 1973. In the following four decades there have been sporadic reports in the medical literature of children with T9 [10] or T9 mosaicism associated with variable levels of somatic mosaicism ranging from 3 % to 99 % [11]. Children born with T9 usually die following birth or within one year of birth whereas those born with T9 mosaicism can survive into adulthood.

Based on data submitted to the tracking rare incidence syndromes (TRIS) project [11] and data published by the Rare Chromosomal Disorder Support Group (www.rarechromo.org), T9 mosaicism is generally associated with a low birth weight and a wide spectrum of developmental disabilities, abnormal physical features and organ pathologies, although the overall disease phenotype can vary widely between individuals. The main developmental disabilities reported are slow body growth, learning difficulties and delayed communication and speech. Characteristic physical features include microcephaly, loose joints, craniofacial abnormalities such as low set ears, cleft palate as well as skeletal abnormalities of the skull, hands and feet. Multiple tissue pathologies are common and can involve different organ systems such as the heart, kidneys, brain and CNS, the genital and urinary tract, muscles (hypertonia) and digestive tract. In the T9 mosaicism case described in this report, the main disease features displayed by the child were dysmorphic craniofacial features, developmental delay, congenital cerebral dysplasia and congenital heart disease. However, in contrast to other children with a similar low level of T9 mosaicism [11], growth retardation and developmental delay was particularly severe in this 22 month old girl and constant daily care was needed.

Intriguingly, from all the children examined with T9 mosaicism to date, there appears to be no clear correlation of the level of mosaicism with the severity of the disease phenotype [11]. One plausible explanation to reconcile this lack of phenotypic/genotypic correlation is that the level of T9 mosaicism has only generally been assessed in peripheral blood lymphocytes and skin. It is possible that during pre- and post-natal development, the proportion of T9 cells in the various tissues has altered significantly due to differences in the intrinsic growth and apoptotic rates of the normal and trisomic cells. In addition, even within one tissue or organ, regional differences in T9 mosaicism may have also arisen. Three case reports provide evidence to support this notion. In the first case [12], prenatal diagnosis of amniocytes detected 100 % T9 whereas postnatal analysis of kidney, skin, ovary and lung tissue from the deceased child who died at age 6 weeks revealed T9 mosaicism levels of 25 %, 15.4 %, 3.7 % and 0 %, respectively. In two other cases that analysed fetuses that died in utero, there were also discrepancies noted between the level of T9 mosaicism in different tissues [13, 14]. Similarly, in the case presented here, we observed 30 % mosaicism from placental tissue in the prenatal period by NIPT, but only 17 % and 19 % respectively, in blood and buccal cells by interphase FISH.

Extensive data collected from pre-implantation genetic diagnosis cycles [15] demonstrates that chromosomal trisomy arising in the somatic tissue of an individual has an early developmental origin. Several mechanisms have been proposed for the formation of mosaicism including either a gametal meiotic I/meiotic II chromosome non-disjunction error and a subsequent trisomy rescue by anaphase lag in the dividing pre-implantation embryo or mitotic non-disjunction in one of the early cell divisions of the cleavage stage embryo [16]. Through these mechanisms, the formation of trisomy mosaicism in association with uniparental disomy (UPD) is also another possible outcome [17]. Mosaic embryos can continue to develop throughout the pre- and post implantation period leading to the formation of diploid-aneuploid mosaic fetuses. In this case study, Chr9 STR analyses of the family pedigree revealed that the proband had inherited two identical copies of paternal Chr9 (isodisomy 9) and one copy of maternal Chr9 with comparative paternal:maternal Chr9 ratios of approximately 10:1. On this basis, we conclude that the origin of the T9 mosaicism in the child was most likely due to a paternal meiotic II non-disjunction error of Chr9 followed by a post-zygotic T9 rescue event of the maternally inherited Chr9 in the initial cleavage divisions of the developing embryo. We speculate that these two independent Chr9 events in the pre-implantation period ultimately lead to the development of T9 mosaicism in the child, comprising paternal isodisomy 9 in the majority of somatic cells and T9 in the remaining somatic cells.

From the literature, there have several prenatal reports [18, 19] where the fetus has been identified with T9 mosaicism in association with maternal UPD 9. Based on records of all documented UPD 9 cases (http://upd-tl.com/upd.html), there have been 10 births with maternal UPD 9 and one birth with paternal UPD 9. Clinical examination of those individuals with maternal UPD 9, which is associated with maternally imprinted genes, revealed no significant disease phenotypes [20]. However, when the maternal UPD9 was also associated with T9, the patients displayed typical symptoms characteristic of trisomy 9 mosaicism syndrome. The one case of paternal UPD 9 was also found in the context of trisomy 9 mosaicism with the child exhibiting typical clinical symptoms of trisomy 9 mosaicism syndrome [21]. Thus the child reported in this study represents the second individual identified with paternal UPD 9, which we identified as isodisomy 9. Based on the very severe developmental delay exhibited by this child, in addition to the possibility of variable levels of T9 in other tissues, we raise the possibility that recessive genes associated with growth and development on Chr9 such as *DMRT1*, *NTRK2* and *NTRKR2* [22] may have also contributed to the overall clinical disease phenotype of this child.

Over many years traditional prenatal diagnosis by karyotyping and FISH analysis of cultured amniocytes, in combination with ultrasound, has been relatively successful in identifying fetuses with T9 mosaicism [13, 23–25]. However, in clinical practice, decisions regarding termination of pregnancy are extremely difficult because several follow up studies have shown that the level of trisomy detected prenatally is often much lower in post-natal tissue [12, 26]. In addition, it is almost impossible to determine the clinical significance of low-level trisomy in cultured amniocytes since it could represent either true fetal mosaicism or pseudomosaicism as a result of culture artifacts [27]. The case reported here was additionally confounding because the low-level T9 originally detected at 15 weeks gestation by NIPT was not identified by traditional confirmation by amniocentesis, cell culture and karyotyping even at 34 weeks gestation. This suggests that in this case, culturing of amniocytes may have caused preferential growth of normal cells over abnormal cells, masking the low level of T9 mosaicism. This case therefore highlights the need for direct analysis of the biopsied fetal cells without culture to increase detection sensitivity when low-level T9 mosaicism is suspected [28].

## Conclusions

In summary, with increasing numbers of pregnant women undertaking NIPT as the primary test method for common fetal aneuploidies, this study shows the feasibility of also detecting a rare chromosomal disorder such as T9 mosaicism syndrome. Apart from T9 mosaicism, T8 mosaicism can also develop to term and cause severe disease phenotypes [29]. While reporting detection of these two rare chromosomal disorders diseases would extend the clinical utility of NIPT beyond common autosomal trisomies and sex aneuploidies, on a cautionary note, this may in turn increase the false positive rates since T8 and T9 are often associated with confined placental mosaicism [30]. Nevertheless NIPT, in conjunction with confirmatory invasive testing and ultrasound scanning, may ultimately prove a more robust approach to identify fetuses with these rare chromosome disease syndromes.

## Materials and methods

### Noninvasive prenatal testing

Clinical NIPT was performed according to previously described methods [5]. In brief, plasma DNA libraries were generated and subjected to massively parallel sequencing on the HiSeq2000 platform (Illumina, US) to generate ~8 million (M) 36 bp sequencing reads per sample. Using the Burrows Wheeler algorithm [31], ~5 M reads were uniquely and perfectly aligned to the unmasked hg19 reference genome. After normalization and comparison to reference data, z-scores were assigned for each chromosome, with a normal range of −3 < z < 3 for autosomal disomy.

### Cytogenetic studies

Metaphase chromosomes were prepared from cultured PHA-stimulated peripheral blood cells. Karyotypes were determined from G banded metaphase chromosome analysis of 100 cells at a resolution of 320 bands. Interface FISH was performed on peripheral blood and buccal cells using 9p SpectrumGreen (locus 305 J7-T7) and 9q SpectrumOrange (locus D9S325) probes (TelVysion, Abbott Molecular, US) according to the recommended protocol. At least 100 cells were examined with 9p and 9q probes to determine levels of Chr9 mosaicism.

### Quantitative fluorescent PCR analysis

STR markers D9S157, D9S164, D9S288, D9S925, D9S1118, D9S1121, D9S1677, D9S1874 and D9S2157 with high heterozygosity indices (>0.7) were selected for Chr9 analysis, with markers D21S1270 and DS13S325 serving as disomy controls. Forward primers for each STR marker were labeled with either 6-carboxy fluorescein (6FAM) or carboxy tetramethylrhodamine (TAMRA). Singleplex QF-PCR reactions (30 amplification cycles) were performed using 50 ng of genomic DNA (Kapa Taq HotStart PCR kit, Kapa Biosystems) according to the manufacturer’s instructions. Labeled PCR products were analyzed on a 3130 Genetic Analyzer (Applied Biosystems, US) and allelic peaks plotted as relative fluorescence intensity (Y-axis) versus size in nucleotides (X-axis).

## Consent

Written informed consent was obtained from the parents of the child for the publication of this report and any accompanying images.

## Abbreviations

NIPT: Noninvasive prenatal testing
QF-PCR: Quantitative fluorescent polymerase chain reaction
STR: Short tandem repeat
T: Trisomy
UPD: Uniparental disomy
